# Cuproptosis and prostate cancer: from molecular mechanisms and microenvironment remodeling to precision therapy

**DOI:** 10.3389/fonc.2026.1874772

**Published:** 2026-06-17

**Authors:** Zhonghao Tang, Si Shen, Chen Guo, Anjie Chen, Dongjie Yang, Jian Sun, Yuanyuan Mi

**Affiliations:** 1 Department of Urology, Affiliated Hospital of Jiangnan University, Wuxi, Jiangsu, China; 2 Wuxi School of Medicine, Jiangnan University, Wuxi, Jiangsu, China; 3 Department of Pathology, Affiliated Hospital of Jiangnan University, Wuxi, Jiangsu, China

**Keywords:** combination therapy, copper metabolism, cuproptosis, prostate cancer, tumor microenvironment remodeling

## Abstract

Prostate cancer (PCa) is a leading malignancy, and progression to castration-resistant prostate cancer (CRPC) remains a central therapeutic challenge. Cuproptosis, a copper-dependent cell death mechanism first characterized in 2022, is triggered by copper binding to lipoylated tricarboxylic acid (TCA)-cycle proteins, inducing their aggregation, Fe-S cluster protein instability, and mitochondrial proteotoxic stress. This review critically evaluates the emerging but still heterogeneous evidence linking cuproptosis to PCa, explicitly distinguishing prostate cancer-specific data from pan-cancer,non-prostate, bioinformatic, and preclinical observations. We describe the core machinery, including ferredoxin 1 (FDX1), dihydrolipoamide acetyltransferase (DLAT), protein lipoylation enzymes, and copper transport/chaperone systems, while emphasizing differences from apoptosis and ferroptosis. In PCa, altered copper homeostasis and mitochondrial metabolic rewiring provide a biologically plausible vulnerability, but current evidence does not yet establish cuproptosis as a validated clinical driver or therapeutic target. We therefore summarize cuproptosis-related gene expression profiles and prognostic models as hypothesis-generating biomarkers, and provide a prostate cancer-specific evidence table that separates bioinformatic, *in vitro*, *in vivo*, and clinical levels of support. We also review potential links with metabolic reprogramming, immune microenvironment modulation, PD-L1 regulation, androgen receptor signaling, PTEN/PI3K pathway activity, epigenetic regulation, and crosstalk with ferroptosis. Therapeutically, copper ionophores such as elesclomol and disulfiram/copper, and copper-depleting approaches such as chelators, are discussed as investigational strategies rather than near-clinical solutions. Particular attention is given to toxicity, narrow therapeutic windows, negative or inconclusive clinical data, the absence of validated companion diagnostics in PCa, and the need for patient-selection biomarkers based on copper handling, FDX1/lipoylation status, and mitochondrial dependency. Finally, we outline the experimental and translational studies required before cuproptosis-directed interventions can be rationally tested in advanced PCa.

## 1 Introduction

Prostate cancer (PCa), a malignancy that poses a severe threat to the health of middle-aged and elderly men worldwide, has seen its incidence and mortality rates continuously rise globally, constituting an increasingly serious public health challenge (1). Although patients with early-stage localized disease can achieve favorable outcomes through radical prostatectomy or radiotherapy, for advanced or metastatic prostate cancer, androgen deprivation therapy (ADT) remains the cornerstone of systemic treatment (2). However, virtually all patients receiving ADT will ultimately and inevitably progress to castration-resistant prostate cancer (CRPC). In the CRPC stage, tumor cells shed their dependence on testicular androgens and become insensitive to conventional endocrine therapy, leading to an extremely poor prognosis. The efficacy of subsequent chemotherapy (e.g., docetaxel) and novel hormonal agents (e.g., abiraterone, enzalutamide) is also relatively limited, and acquired resistance constitutes the core bottleneck in clinical management (3). Consequently, deeply exploring the unique biological characteristics and molecular vulnerabilities of prostate cancer, particularly CRPC, and identifying and validating entirely new therapeutic targets and strategies represent the most urgent tasks in current prostate cancer research.

Cell death is a fundamental physiological process for maintaining organismal homeostasis and eliminating damaged or malignantly transformed cells, and precisely inducing tumor cell death is the common goal of nearly all anticancer therapies. Over the past decades, our understanding of the molecular mechanisms of various forms of programmed cell death (PCD), such as apoptosis, necroptosis, pyroptosis, and ferroptosis, has been continuously deepened (4). Each mode of PCD is exquisitely regulated by distinct molecular pathways, offering a wealth of potential targets for cancer therapy. Among them, ferroptosis, an iron-dependent form of cell death driven by lipid peroxidation, has garnered substantial attention in prostate cancer research in recent years and has demonstrated immense potential in overcoming therapeutic resistance (5). However, the inherent heterogeneity and robust adaptive evolutionary capacity of tumor cells enable them to evade single or multiple cell death pathways through various mechanisms, continuously driving scientists to discover yet-underappreciated cell death mechanisms.

Against this backdrop, in 2022, Tsvetkov and colleagues published a seminal study in Science that systematically defined and elucidated a novel mode of cell death directly mediated by copper ions—cuproptosis (6). The core mechanism of cuproptosis is that excessive intracellular copper ions directly bind with high affinity to lipoylated proteins within the mitochondrial tricarboxylic acid (TCA) cycle, causing the irreversible oligomerization and aberrant aggregation of these key metabolic enzymes. This protein aggregation subsequently triggers the rapid degradation of iron-sulfur (Fe-S) cluster-containing proteins, ultimately inducing a unique form of proteotoxic stress and leading to the collapse of the mitochondrial respiratory chain and cell death (7). This mechanism exhibits uniqueness at multiple levels: it is independent of the canonical caspase cascade activation, distinct from the lipid peroxidation process of ferroptosis, and not mediated by mixed lineage kinase domain-like protein (MLKL) in necroptosis, thereby providing a completely novel theoretical framework and targeting dimension for understanding how cells respond to metal ion toxicity and how to harness this process for disease treatment (8).

Copper, an essential trace element, serves as an obligatory cofactor for enzymes such as cytochrome c oxidase, superoxide dismutase 1 (SOD1), and lysyl oxidase (LOX), and is involved in cellular respiration, antioxidant defense, iron metabolism, and extracellular matrix remodeling (9). Many tumors, including PCa, show alterations in copper content or copper-handling pathways; however, the magnitude, direction, and clinical implications of these changes vary across cohorts and assay platforms, and should not be interpreted as uniformly proving a copper-dependent oncogenic state. The term tumor “copper addiction” is useful as a conceptual framework, but in PCa it should be applied cautiously: the strongest prostate-specific evidence links copper transport and copper-dependent signaling to tumor biology rather than directly proving endogenous cuproptosis. For example, androgen receptor (AR) signaling has been reported to enhance CTR1/SLC31A1-dependent copper uptake in PCa models, thereby connecting a lineage-defining oncogenic pathway with copper homeostasis (10). This provides a rationale for studying copper perturbation in PCa, but it does not by itself demonstrate that PCa cells can be selectively killed through cuproptosis in patients. Pharmacological elevation of intracellular copper by ionophores may trigger cuproptosis in suitably vulnerable cells, whereas copper chelation may inhibit copper-dependent tumor-supportive processes such as LOX activity; both strategies require careful evaluation of context, dose, systemic toxicity, and biomarkers before clinical translation (11, 12).

Given that prostate cancer cells can exhibit copper metabolic abnormalities (13) and that mitochondrial metabolism and TCA-cycle activity contribute to disease progression and CRPC evolution (14), cuproptosis is a mechanistically relevant framework for PCa research. Nevertheless, the current prostate cancer literature remains uneven. Several studies are based on retrospective bioinformatics analyses of cuproptosis-related genes (CRGs), while direct functional evidence in PCa cell lines, organoids, xenografts, or clinical specimens is comparatively limited. Therefore, cuproptosis should be presented as a promising but incompletely validated vulnerability rather than as an established therapeutic axis. In this review, we specifically separate PCa-specific findings from extrapolations derived from other tumor types, and we highlight the experimental steps required to move from association to causation.

Therefore, at this juncture in 2026, it is particularly important and urgent to systematically review and deeply analyze the latest advances in cuproptosis within the field of prostate cancer research. Based on the most recent literature, this review will first systematically elaborate on the molecular machinery of cuproptosis and its regulatory network, and then delve into its multidimensional roles in prostate cancer progression, including regulating tumor cell behavior, remodeling the TME, and crosstalk with other key biological processes. We will further highlight the upstream signaling pathways and epigenetic mechanisms that govern cuproptosis sensitivity in prostate cancer, and ultimately, comprehensively evaluate the current preclinical research status, combination therapy potential, and future prospects and challenges for clinical translation of therapeutic strategies targeting cuproptosis (including copper ionophores and chelators). Through this review, we aim to provide researchers in this field with a comprehensive and in-depth knowledge landscape, and to offer new insights and theoretical foundations for developing innovative therapies against prostate cancer, especially refractory CRPC.

## 2 Molecular mechanisms of cuproptosis

The discovery of cuproptosis has invigorated the field of cell death research, and its unique molecular mechanism serves as the cornerstone for understanding its role in various diseases, particularly cancer. This process begins with the sensing of an imbalance in intracellular copper ion concentrations and ultimately strikes at the core of mitochondrial metabolism through a precise and lethal cascade. This chapter will detail the discovery process of cuproptosis, its core molecular events, key regulators, and the upstream cellular copper homeostasis regulatory network, thereby constructing a complete molecular blueprint of cuproptosis.

### 2.1 Discovery and core characteristics of cuproptosis

**Figure 1:**
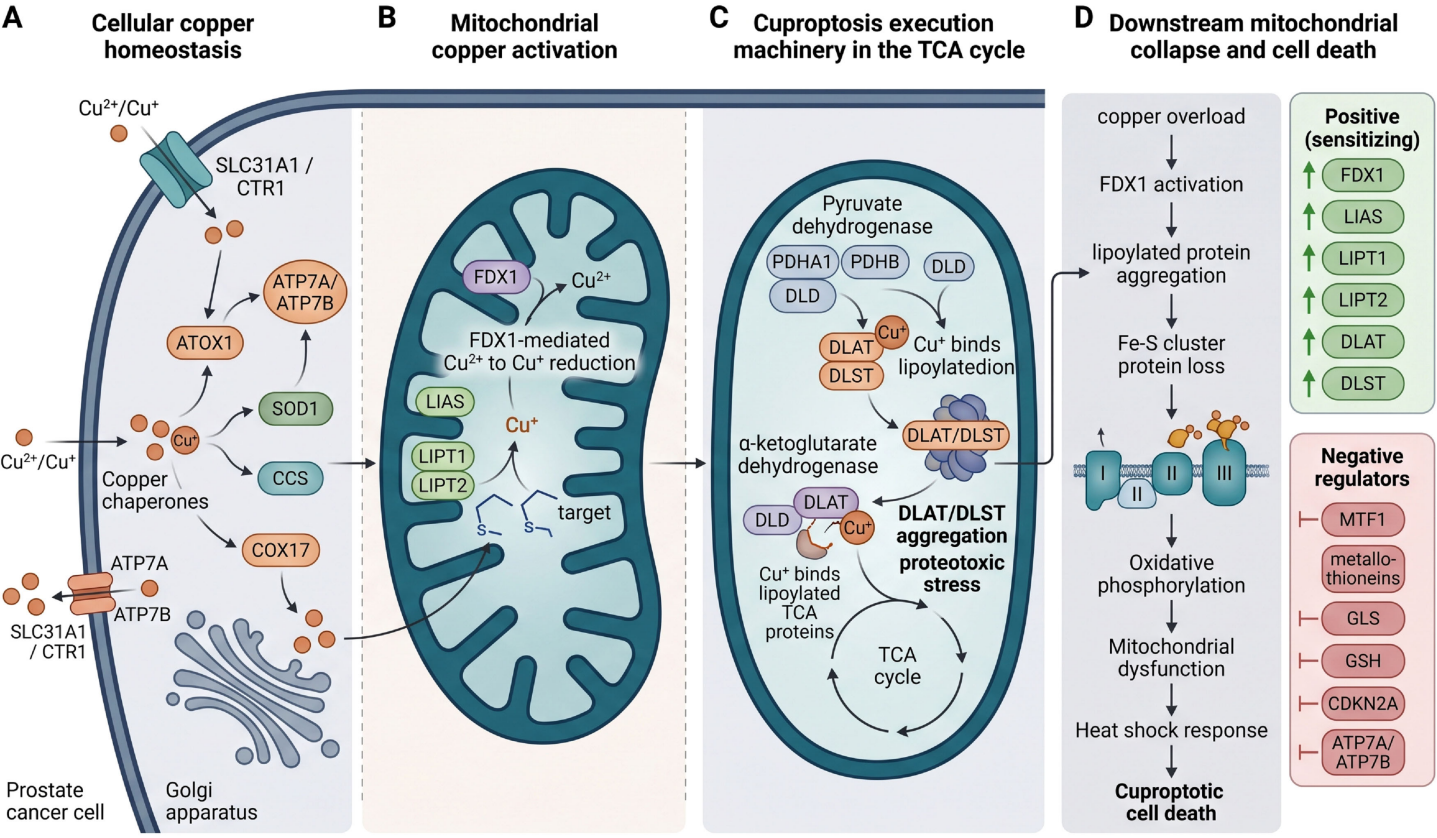
Schematic overview of the molecular mechanism of cuproptosis. **(A)** Cellular copper homeostasis is maintained by influx via SLC31A1 (CTR1), intracellular distribution by copper chaperones, and efflux via ATP7A/B. **(B)** Within the mitochondria, FDX1 mediates the reduction of Cu²^+^ to the highly reactive Cu^+^, parallel to the lipoylation of target proteins driven by LIAS, LIPT1, and LIPT2. **(C)** Cu^+^ directly binds to lipoylated TCA cycle proteins (primarily DLAT and DLST), triggering their abnormal aggregation and inducing proteotoxic stress. **(D)** This aggregation initiates a downstream cascade characterized by the destabilization of Fe-S cluster proteins, impairment of the mitochondrial respiratory chain, and ultimately, cuproptotic cell death. The right panel summarizes key positive (sensitizing) and negative (suppressive) regulators of the cuproptosis pathway.

The formal establishment of cuproptosis as an independent cell death program originated from in-depth investigations into the anticancer mechanisms of copper ionophores such as elesclomol. For a long time, scientists had observed that high concentrations of copper exert significant cytotoxicity, yet the precise lethal mechanism remained an enigma, often vaguely attributed to the generation of reactive oxygen species (ROS). However, in their landmark 2022 study, Tsvetkov and colleagues systematically unveiled this mystery by combining loss-of-function genome-wide CRISPR-Cas9 screening with sophisticated metabolomic analyses (6). They discovered that cell lines highly sensitive to copper ionophores uniformly exhibited a strong dependence on mitochondrial respiration. The pivotal CRISPR screen precisely identified several genes associated with protein lipoylation and iron-sulfur (Fe-S) cluster biogenesis, the loss of which conferred significant resistance to copper ionophores. Based on this robust genetic and biochemical evidence, they formally named this copper-dependent, mitochondrial respiration-regulated, and proteotoxic stress-centered form of cell death “cuproptosis.” The core characteristic of cuproptosis lies in its unique triggering mechanism: it is not driven by the apoptotic caspase cascade, lipid peroxidation (ferroptosis), or receptor-interacting protein kinase (RIPK) activation (necroptosis), but rather stems from a copper-induced proteotoxic stress that targets specific proteins (**Figure 1**). This mechanistic specificity fundamentally distinguishes it from all known cell death modalities at the molecular level, thereby carving out a completely new branch in the cell death spectrum (15).

### 2.2 Key molecular pathway of cuproptosis

The molecular pathway of cuproptosis is intimately centered on a core metabolic hub within the mitochondrial matrix—the TCA cycle. The TCA cycle is not only the primary site of aerobic respiration for ATP production but also features several multienzyme complexes, particularly the pyruvate dehydrogenase complex (PDC) and the α-ketoglutarate dehydrogenase complex (OGDHC), which contain an essential coenzyme, lipoic acid. Lipoic acid is covalently attached via its carboxyl group to the ϵ-amino group of specific lysine residues on the E2 subunits of these enzyme complexes, a modification process known as lipoylation (16). Lipoylated proteins, especially their dithiolane ring moiety, play an indispensable “swinging arm” role in catalyzing substrate oxidative decarboxylation and acyl group transfer. The initiating step of cuproptosis occurs when elevated intracellular Cu²^+^ ions are reduced to the more chemically reactive and cytotoxic Cu^+^ form by ferredoxin 1 (FDX1), a mitochondrial inner membrane reductase (17). Subsequently, the newly generated Cu^+^ binds with exceptionally high affinity directly to the lipoyl moieties of TCA cycle lipoylated proteins, primarily the E2 subunit of PDC, dihydrolipoamide acetyltransferase (DLAT), and the E2 subunit of OGDHC, dihydrolipoamide succinyltransferase (DLST) (18). This direct copper-lipoic acid binding disrupts the normal conformation and function of these proteins, causing their aberrant oligomerization and aggregation into insoluble protein clumps, thereby obstructing the entire TCA cycle metabolic flux (19). This abnormal protein aggregation constitutes the initiating event of proteotoxic stress and rapidly triggers downstream cascades. Following this, the aggregated lipoylated proteins further destabilize Fe-S cluster proteins. Fe-S clusters are ubiquitous cofactors in many crucial proteins, including mitochondrial respiratory chain complexes I, II, and III, aconitase in the TCA cycle, and numerous DNA repair enzymes, and are essential for maintaining their structure and catalytic activity (20). The aggregation of lipoylated proteins leads, through incompletely understood mechanisms, to the rapid degradation and loss of these Fe-S cluster proteins, severely impairing mitochondrial electron transport chain function and overall cellular metabolic homeostasis. Ultimately, the heat shock response elicited by proteotoxic stress and Fe-S cluster protein loss is insufficient to rescue the cell, leading to irreversible cell death. Thus, from upstream copper ion accumulation and reduction, to the core aggregation of lipoylated proteins, and to the downstream loss of Fe-S cluster proteins, together they constitute a linear, interlocking molecular pathway of cuproptosis, with its core battlefield in the mitochondrial matrix (21).

### 2.3 Core regulators of cuproptosis

The execution of cuproptosis is akin to a precise molecular lock, the opening and closing of which are exactly controlled by a suite of genes and proteins that can be clearly divided into positive and negative regulators based on their functions. Positive regulators are molecules that promote cuproptosis; their enhanced expression or activity sensitizes cells to copper toxicity. Among them, FDX1 is recognized as the “upstream master switch” of the cuproptosis pathway (22). Ferredoxin 1 (FDX1) is a [2Fe-2S] cluster protein localized in the mitochondria—its canonical function is involvement in steroid hormone biosynthesis, but in the cuproptosis pathway, it acts as the catalyst for the critical chemical step of reducing Cu²^+^ to Cu^+^ (23). A specific structural domain, such as α-helix 3, is essential for this function (24). Therefore, the expression level of FDX1 directly determines the rate of “ammunition” generation for catalyzing cuproptosis; low expression or loss of function of FDX1 is one of the core mechanisms by which tumor cells evade cuproptosis and develop drug resistance (25). Besides FDX1, members of the entire protein lipoylation synthesis pathway are also crucial positive regulators. Lipoic acid synthase (LIAS) is responsible for the *de novo* synthesis of lipoic acid; lipoyltransferase 1 and 2 (LIPT1/2) are responsible for activating the synthesized lipoic acid and covalently attaching it to the E2 subunits of dehydrogenase complexes (26). Experimental evidence indicates that FDX1, through direct interaction with LIAS, coordinately regulates the lipoylation level of DLAT, jointly forming an upstream signaling module of cuproptosis. Additionally, as direct targets of copper ions, the normal expression and assembly of multiple subunits of the PDC and OGDHC complexes, such as DLAT, DLD, PDHA1, and PDHB, are prerequisites for cuproptosis to occur (27). This suite of genes constitutes the execution scaffold of cuproptosis; dysfunction at any link will interrupt signal transduction. Negative regulators are molecules that inhibit cuproptosis, forming the cellular defense system against copper toxicity. The metallothionein (MT) family comprises low-molecular-weight proteins rich in cysteine that can efficiently bind and chelate excess heavy metal ions, including copper; they act like “molecular sponges,” absorbing free copper ions and thereby preventing their binding to target proteins such as DLAT, making them one of the most important copper detoxification proteins. Metal-regulatory transcription factor 1 (MTF1) is the key transcription factor governing MT gene expression, activated under copper stress to rapidly upregulate MT expression, thus suppressing cuproptosis. Moreover, some proteins involved in metabolic regulation have also been found to negatively regulate cuproptosis. For instance, glutaminase (GLS), by promoting glutamine decomposition, provides raw material for the synthesis of glutathione (GSH), the most important intracellular antioxidant and copper chelator, thereby antagonizing cuproptosis. Cyclin dependent kinase inhibitor 2A (CDKN2A) has also been identified as a negative regulator, although its specific inhibitory mechanism awaits further elucidation (26).

### 2.4 Regulation of cellular copper homeostasis

The prerequisite for triggering cuproptosis is that the concentration of bioavailable copper ions exceeds the physiological homeostatic threshold. Therefore, understanding how cells maintain copper homeostasis through a complex and sophisticated system is critical to grasping the physiological and pathological significance of cuproptosis. Cells employ an efficient “import-efflux-distribution” system to keep the concentration of free copper ions in the cytosol at an extremely low (femtomolar) and safe range. Copper import is primarily the responsibility of the high-affinity copper transporter 1 (CTR1; gene name SLC31A1) located on the plasma membrane. SLC31A1 exists as a trimer and efficiently transports Cu^+^, reduced by a cell-surface reductase, into the cell (28). The expression level of SLC31A1 directly determines the total amount of copper taken up by the cell, thus serving as a crucial “entry” valve dictating cellular sensitivity to cuproptosis. Copper export is mainly mediated by two types of P-type ATPases—ATP7A and ATP7B. They are typically localized in the trans-Golgi network, responsible for pumping excess copper ions from the cytoplasm into the Golgi lumen for the synthesis of cuproenzymes. When intracellular copper concentrations become excessive, ATP7A and ATP7B undergo dynamic trafficking from the Golgi to the plasma membrane or vesicular membranes to actively pump copper out of the cell, acting as “detoxification pumps” (29). Functional defects in ATP7A or ATP7B (as seen in Wilson’s disease and Menkes disease) lead to catastrophic intracellular copper accumulation, greatly increasing the risk of cuproptosis. Within the cell, the safe distribution of copper is executed by a series of dedicated copper chaperones. For example, ATOX1 acts as an “escort,” receiving copper from SLC31A1 and safely delivering it to ATP7A/B in the Golgi; CCS is responsible for delivering copper to cytoplasmic copper/zinc superoxide dismutase (SOD1); and COX17 and its downstream partners deliver copper to the mitochondria for the assembly of respiratory chain complex IV (cytochrome c oxidase) (30). This intricate distribution network ensures that copper is safely and efficiently delivered to functional sites while strictly avoiding free copper ion-catalyzed harmful Fenton-like reactions that generate ROS. In summary, the molecular mechanism of cuproptosis is a multi-step cascade initiated by upstream copper homeostatic imbalance, with FDX1-mediated copper ion reduction as the initiating step, the aggregation of lipoylated proteins in the TCA cycle as the core event, and downstream Fe-S cluster protein loss as an amplification signal, all tightly controlled by a series of positive and negative regulators. It is this unique and clear molecular pathway that provides unprecedented opportunities for understanding and intervening in the progression of diseases such as prostate cancer.

## 3 Roles and regulation of cuproptosis in prostate cancer progression

**Figure 2:**
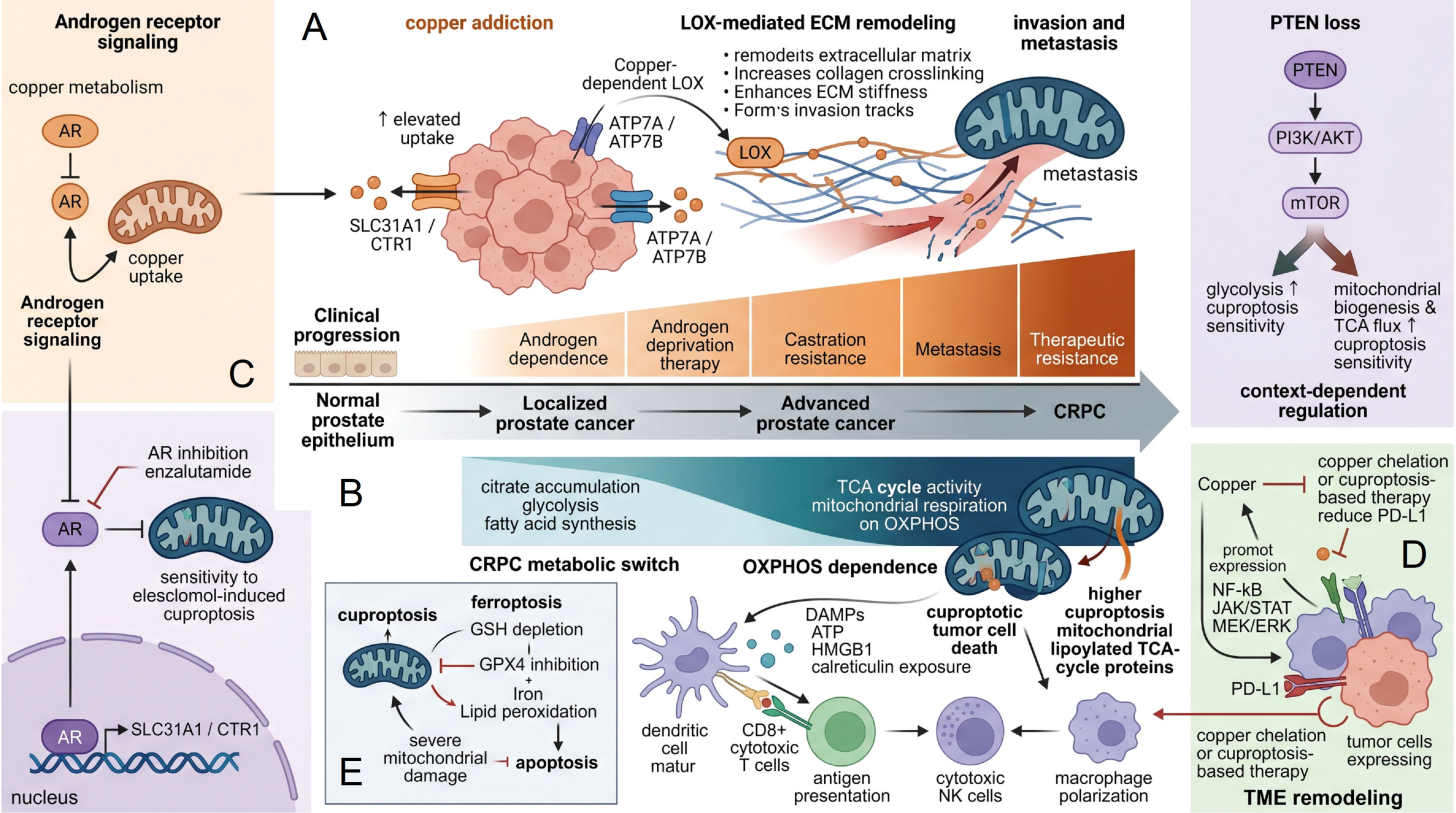
The multidimensional role of cuproptosis in prostate cancer progression and therapeutic vulnerability. The central axis illustrates the clinical progression from normal epithelium to castration-resistant prostate cancer (CRPC), driven by increasing copper addiction. **(A)** Elevated intracellular copper promotes LOX-mediated extracellular matrix (ECM) remodeling, facilitating invasion and metastasis. **(B)** Metabolic reprogramming in CRPC toward oxidative phosphorylation (OXPHOS) creates an enhanced dependency on mitochondrial function, heightening vulnerability to cuproptosis. **(C)** Copper metabolism and cuproptosis sensitivity are regulated by AR and PTEN/PI3K/AKT/mTOR signaling networks. **(D)** Cuproptotic cell death remodels the tumor immune microenvironment (TIME) by releasing DAMPs to activate immune cells, while copper signaling regulates PD-L1 expression. **(E)** Cuproptosis exhibits mechanistic crosstalk with ferroptosis and apoptosis, linked primarily through glutathione (GSH) depletion and severe mitochondrial damage.

The development and progression of prostate cancer is a multi-step, multi-gene process in which metabolic reprogramming and evasion of cell death are central malignant features. Because cuproptosis is mechanistically linked to mitochondrial metabolism, it may be relevant to PCa biology; however, the strength of evidence differs markedly across topics. At present, many associations between CRGs and prognosis or immune infiltration are derived from public transcriptomic datasets, whereas direct prostate cancer experiments remain relatively sparse. Accordingly, the following sections evaluate cuproptosis as a testable hypothesis and potential vulnerability, not as a fully established driver of PCa progression (**Figure 2**).

### 3.1 Copper homeostasis disturbance and prostate cancer

Copper acts as an exquisite “double-edged sword” (31) and its homeostatic imbalance may contribute to tumorigenesis and progression (32). Compared with normal tissues or healthy controls, some studies have reported altered copper levels in prostate cancer tissues or serum, and associations with higher Gleason score, stage, metastasis, or biochemical recurrence risk. However, these observations are correlative and may be influenced by tumor burden, inflammation, sampling method, and analytical platform; they should therefore be interpreted as supportive rather than definitive evidence for a causal copper-driven phenotype. PCa cells may alter copper uptake or efflux through transporters such as SLC31A1/CTR1 and ATP7A/B, and AR activity can modulate copper uptake through SLC31A1/CTR1-dependent mechanisms. Such findings provide a mechanistic link between prostate lineage signaling and copper handling, but they do not prove that spontaneous cuproptosis occurs during PCa progression. Similarly, the role of copper-dependent LOX family proteins in extracellular matrix remodeling and metastasis supports the broader importance of copper biology (33), while remaining distinct from canonical cuproptosis. Therapeutic perturbation of copper homeostasis may therefore influence PCa through multiple mechanisms, including cuproptosis induction, inhibition of cuproenzymes, oxidative stress, proteasome inhibition, or microenvironment remodeling. Future studies should quantify labile copper pools, FDX1 activity, protein lipoylation, DLAT/DLST aggregation, and Fe-S cluster protein loss in PCa models to demonstrate true cuproptosis rather than nonspecific copper toxicity.

### 3.2 Expression profile and clinical significance of cuproptosis-related genes in prostate cancer

Following elucidation of the cuproptosis pathway, multiple groups have mined TCGA, GEO, and related datasets to evaluate CRG expression patterns in prostate cancer (34, 35). These studies have identified cuproptosis-associated molecular subtypes, risk scores, and immune-infiltration patterns, and several models report associations with biochemical recurrence, disease-free survival, clinical stage, Gleason score, or predicted treatment sensitivity (36–39). Prostate cancer-specific analyses have linked CRG-based subtypes or signatures to the tumor microenvironment and prognosis. These results are valuable for hypothesis generation, but they should not be overinterpreted as validated clinical biomarkers. Most models were developed retrospectively, often using overlapping public cohorts, variable CRG definitions, and computational immune-deconvolution methods; many lack independent prospective validation, standardized assay cutoffs, and functional confirmation. Furthermore, mRNA abundance of FDX1, DLAT, LIAS, DLD, or related genes does not necessarily reflect mitochondrial copper availability, protein lipoylation, lipoylated-protein aggregation, or Fe-S cluster instability. Thus, CRG signatures should currently be considered exploratory biomarkers that require validation in independent PCa cohorts, patient-derived organoids, xenografts, and clinical specimens with matched treatment outcomes. (**Table 1**).

**Table 1:** Prostate cancer-specific evidence relevant to copper metabolism, cuproptosis-related genes, and cuproptosis-targeting strategies.

Study/source	Model system	Key copper/cuproptosis-related genes or pathways	Drug/intervention	Main findings	Evidence type and key limitations
Safi et al., 2014 (33)	PCa cell and preclinical models	AR signaling; CTR1/SLC31A1-dependent copper uptake; copper signaling	Copper manipulation; copper-targeting concepts	AR activity was linked to increased copper uptake, providing a prostate-specific rationale for studying copper handling in PCa.	*In vitro*/preclinical; predates formal definition of cuproptosis and does not prove canonical cuproptosis.
Jin et al., 2022 (37)	TCGA/GEO prostate cancer transcriptomic datasets	CRG-based subtypes and prognostic signature	None	Cuproptosis-related subtypes and risk model were associated with prognosis and tumor microenvironment infiltration.	Bioinformatic; retrospective; requires prospective validation and functional confirmation.
Li et al., 2024 (36)	Public PRAD cohorts	FDX1, DLAT, LIAS, DLD, CDKN2A and related CRGs	None	CRG expression patterns were linked to prognosis, immune microenvironment features, and predicted therapeutic sensitivity.	Bioinformatic; no direct proof of cuproptosis execution in PCa tissue.
Yao et al., 2023 (4)	PRAD transcriptomic datasets	Cuproptosis-related immune genes	None	An immune-related cuproptosis signature was proposed to predict prognosis and tumor microenvironment characteristics.	Bioinformatic; immune deconvolution and retrospective modeling need independent validation.
Wang et al., 2023 (27)	TCGA/GEO prostate cancer datasets	Hub CRGs identified for PCa development prediction	None	A cuproptosis-related gene signature was proposed for predicting PCa development.	Bioinformatic; diagnostic/prognostic value needs experimental and clinical validation.
Lei et al., 2022 (40)	PCa cell lines including PC3 and DU145	Copper-dependent disulfiram effects; chloride currents; apoptosis/tyrosine kinase-related pathways	Disulfiram plus copper	Disulfiram/copper produced cytotoxic and apoptosis-related effects in PCa cells.	*In vitro*; mechanisms were not defined as canonical cuproptosis because DLAT aggregation and Fe-S cluster loss were not the central endpoints.
Gao et al., 2024 (41)	CRPC cell lines, enzalutamide-resistant models, and 22Rv1 xenografts	Mitochondrial dependency; lipoylated proteins; Fe-S cluster proteins; cuproptosis hallmarks	Enzalutamide combined with elesclomol or disulfiram/copper	AR blockade increased mitochondrial dependency and sensitized CRPC models to copper ionophores with cuproptosis-associated biochemical features.	*In vitro* and *in vivo* preclinical; no clinical efficacy or validated patient-selection biomarkers.
Zhang et al., 2022 (42)	Patients with metastatic CRPC in a phase Ib trial	Copper handling and disulfiram/copper pharmacodynamics	Oral disulfiram plus copper	The study did not establish meaningful clinical benefit and highlighted toxicity/pharmacokinetic limitations.	Clinical phase Ib; small, non-definitive, negative/inconclusive for efficacy; no validated companion diagnostics.

### 3.3 Cuproptosis regulates prostate cancer cell proliferation, invasion, and metastasis

Cuproptosis is a terminal cell fate, but the genes and copper-handling systems that determine cuproptosis sensitivity may also intersect with proliferation, invasion, and metastasis. In PCa, however, direct causal evidence remains limited. Downregulation of FDX1 or alterations in protein lipoylation could theoretically reduce cuproptosis sensitivity and reshape mitochondrial metabolism, but this has not yet been systematically proven with FDX1/DLAT gain- and loss-of-function experiments across androgen-sensitive, AR-independent, neuroendocrine, and treatment-resistant PCa models. Evidence from other tumor types suggests that FDX1 can suppress malignant phenotypes through DLAT lipoylation and cuproptosis induction, but such findings should be described as extrapolations rather than prostate cancer-specific proof. Similarly, sublethal copper stress may activate heat-shock, Nrf2, metallothionein, or glutathione-based adaptive responses that protect tumor cells and contribute to therapy resistance; whether these mechanisms operate during PCa treatment remains to be tested. Existing PCa-specific experimental data mainly support copper-dependent cytotoxicity or growth inhibition rather than a complete, canonical cuproptosis mechanism. For instance, disulfiram/copper was reported to induce cytotoxic and apoptotic effects in PCa cell lines, including PC3 and DU145, but that study emphasized chloride currents and tyrosine kinase-related apoptosis rather than DLAT aggregation or Fe-S cluster loss (40). More direct support comes from recent CRPC models showing that enzalutamide can increase mitochondrial dependency and sensitize cells to copper ionophores with cuproptosis hallmarks, including lipoylated protein aggregation and Fe-S cluster protein instability (41). Overall, these findings justify further study of cuproptosis in PCa proliferation and metastatic biology, but they do not yet establish cuproptosis as a dominant endogenous regulator of invasion or metastasis.

## 4 Interplay between cuproptosis and other key biological processes

Cuproptosis is not an isolated biological event; it is deeply embedded in the complex network of cellular activities, intertwining with and mutually influencing a variety of key biological processes within and outside tumor cells, forming a dynamic and intricate regulatory network. A profound understanding of these interactions is paramount for comprehensively appreciating the role of cuproptosis in prostate cancer and for designing effective, synergistic combination therapeutic strategies.

### 4.1 Cuproptosis and tumor metabolic reprogramming

Cuproptosis is mechanistically coupled to tumor metabolic state because its execution depends on mitochondrial copper accumulation, protein lipoylation, and an active TCA/OXPHOS axis. In this setting, DLAT and DLST are not simply metabolic enzymes; they are lipoylated mitochondrial targets. Excess copper binds lipoylated TCA-cycle components, promotes their aberrant oligomerization/aggregation, destabilizes Fe-S cluster proteins, and generates mitochondrial proteotoxic stress. Consequently, mitochondrial respiration is disrupted and OXPHOS is progressively impaired, rather than being nonspecifically or instantaneously “strangled” (6, 43).

This mitochondria-centered mechanism explains why the metabolic evolution of prostate cancer is relevant to cuproptosis sensitivity. Normal prostate epithelium, and some localized lesions that retain lineage metabolic features, are characterized by a citrate-secretory phenotype: high zinc levels inhibit mitochondrial aconitase, truncate the TCA cycle, and favor citrate accumulation and secretion. During malignant progression, reduced zinc accumulation and restoration of aconitase activity permit citrate oxidation, reactivation of a more complete TCA cycle, and increased mitochondrial use of carbon substrates for ATP production and lipogenesis. In advanced disease and CRPC, this rewiring becomes heterogeneous but often includes enhanced TCA/OXPHOS activity, increased use of fatty-acid and amino-acid substrates, and therapy-induced mitochondrial adaptation. Therefore, the transition from citrate secretion toward citrate utilization and OXPHOS dependency provides a metabolic bridge between prostate cancer progression and potentially increased vulnerability to cuproptosis (14, 44–46).

AR signaling also intersects with this vulnerability. AR regulates metabolic programs that include lipid synthesis, nutrient transport, mitochondrial pyruvate entry, TCA-cycle inputs, and respiratory activity. In AR-driven prostate cancer, mitochondrial pyruvate import has been identified as a metabolic vulnerability, supporting the concept that mitochondrial substrate flux is functionally linked to AR biology (47). In CRPC cells exposed to enzalutamide, adaptive metabolic rewiring may increase reliance on mitochondrial substrate oxidation and TCA/OXPHOS flux. Gao et al. reported that enzalutamide increased mitochondrial dependence and sensitized CRPC cells to copper ionophores, including elesclomol and disulfiram; the response showed canonical cuproptosis hallmarks, including lipoylated protein aggregation and Fe-S cluster protein instability, and was reversed by copper chelation. Thus, AR blockade should be framed as creating or amplifying an OXPHOS/cuproptosis vulnerability through compensatory mitochondrial rewiring, rather than as a direct cuproptosis inducer.

The inverse relationship is equally important. Tumors that maintain a strongly glycolytic Warburg phenotype may be less sensitive to cuproptosis because they are less dependent on mitochondrial respiration and lipoylated TCA-cycle proteins. Direct experimental evidence supporting this concept comes from the original cuproptosis study, in which cells forced to rely on mitochondrial respiration by galactose substitution were markedly more sensitive to elesclomol-copper than cells relying predominantly on glycolysis (6, 43). More recent literature further supports the idea that glucose-driven glycolysis, hypoxia, and HIF-1α signaling can suppress cuproptosis sensitivity in solid tumors (48, 49). For prostate cancer, this implies that PTEN loss-, hypoxia-, or therapy-associated glycolytic states may blunt the effect of copper ionophores unless combination strategies restore mitochondrial dependency or overcome glycolytic resistance.

Glutamine metabolism should also be considered as more than a biosynthetic pathway. GLS converts glutamine to glutamate, which supplies carbon and nitrogen for anaplerosis and also provides a precursor for GSH synthesis. GSH protects cells through two mechanistically distinct but complementary functions. First, as an antioxidant, GSH maintains thiol-redox homeostasis and limits ROS-associated mitochondrial damage during copper stress. Second, as a copper-buffering molecule, GSH can directly coordinate labile copper and reduce the pool of copper available to engage mitochondrial lipoylated proteins. This distinction is important because GSH depletion may sensitize cells to cuproptosis both by weakening antioxidant defenses and by reducing copper chelation. Recent evidence that NFE2L2/SLC25A39-driven GSH metabolism promotes cuproptosis resistance further supports the copper-buffering role of GSH, in addition to its broader antioxidant function (50, 51).

**Figure 3:**
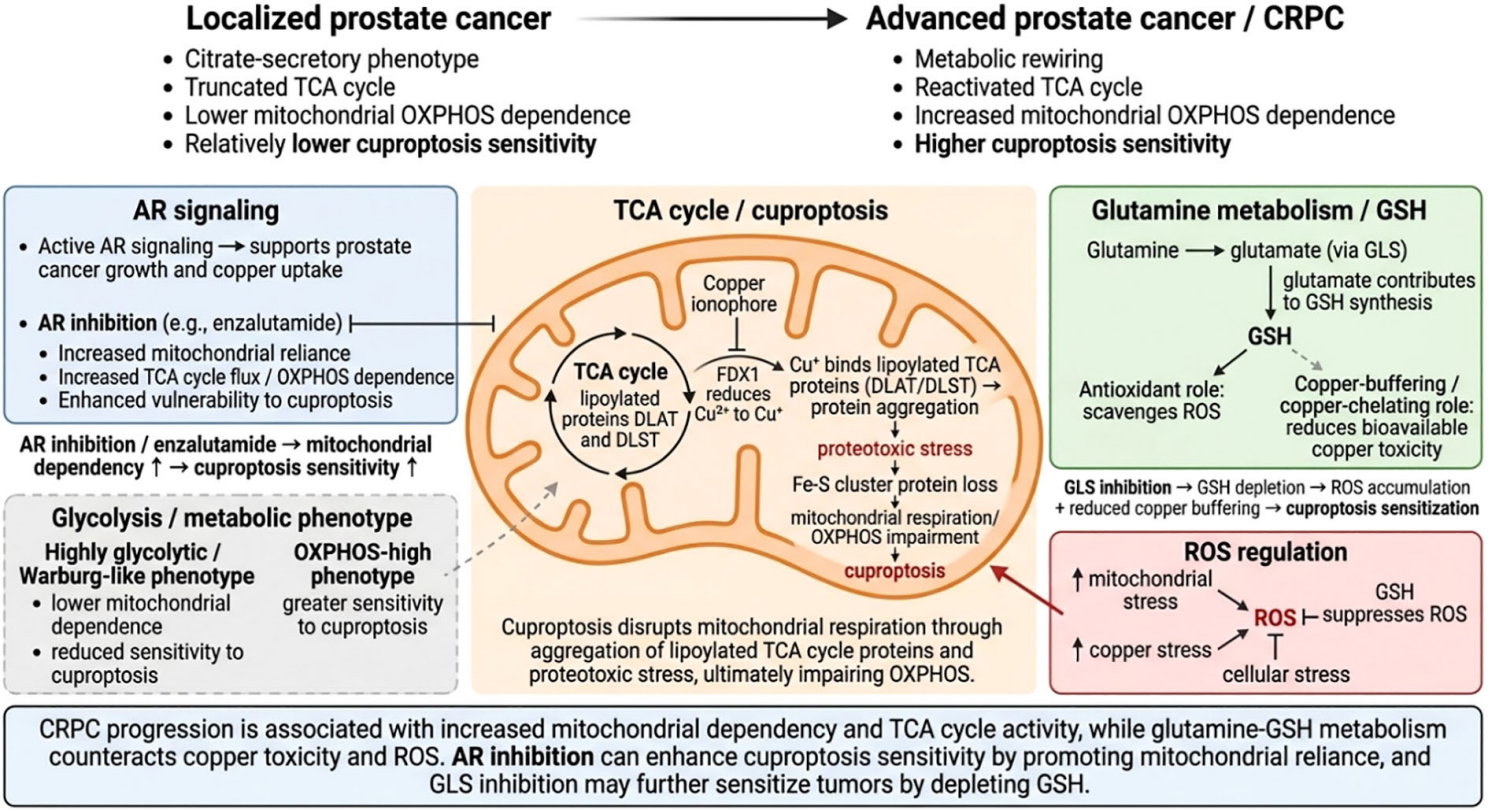
Metabolic rewiring dictates cuproptosis sensitivity during prostate cancer progression. Localized prostate cancer exhibits a truncated TCA cycle and low cuproptosis sensitivity. Progression to castration-resistant prostate cancer (CRPC) involves reactivated TCA cycle activity and increased reliance on mitochondrial oxidative phosphorylation (OXPHOS), heightening tumor vulnerability to cuproptosis. Mechanistically, FDX1 reduces intracellular copper to Cu^+^, which binds lipoylated TCA cycle proteins (DLAT/DLST), causing protein aggregation, proteotoxic stress, and ultimately OXPHOS impairment. While active AR signaling drives tumor growth, AR inhibition (e.g., enzalutamide) induces a compensatory increase in mitochondrial reliance, further sensitizing cells to cuproptosis. Conversely, glutamine-driven glutathione (GSH) synthesis protects tumors by scavenging reactive oxygen species (ROS) and chelating toxic copper. Consequently, GLS inhibition depletes GSH, exacerbating ROS accumulation and sensitizing tumors to copper toxicity. Overall, highly glycolytic (Warburg-like) phenotypes resist cuproptosis, whereas OXPHOS-dependent phenotypes remain highly susceptible.

On this basis, combining cuproptosis inducers with inhibition of the glutamine-GSH axis is a biologically plausible strategy. However, in prostate cancer, direct preclinical evidence showing synergy between clinically used GLS inhibitors and cuproptosis inducers remains limited. This approach should therefore be presented as a testable synthetic-lethal hypothesis for OXPHOS-dependent CRPC rather than as an established therapeutic strategy. Supporting but indirect evidence exists in non-prostate cancer models: GLS2 inhibition combined with copper reprogrammed TCA metabolism and enhanced cuproptosis-driven radiosensitization in esophageal squamous cell carcinoma *in vitro* and *in vivo (*52). Future prostate cancer studies should determine whether GLS1/GLS2 inhibition reduces GSH-mediated copper buffering, increases mitochondrial copper availability, and synergizes with elesclomol-copper or disulfiram-copper in AR-inhibited, OXPHOS-dependent CRPC models (**Figure 3**).

### 4.2 Cuproptosis and the tumor immune microenvironment

The tumor immune microenvironment (TIME) is a crucial determinant of tumor progression, metastasis, and immunotherapy response. Cuproptosis may influence the TIME, but in PCa this connection remains largely inferential. Although some forms of regulated cell death can release damage-associated molecular patterns (DAMPs) and promote antigen presentation, definitive evidence that canonical cuproptosis constitutes immunogenic cell death in prostate cancer is currently lacking. Therefore, statements that cuproptosis can convert prostate tumors from “cold” to “hot” should be framed as a hypothesis requiring validation in immunocompetent PCa models and human specimens. Bioinformatic analyses have associated CRG signatures with immune-cell infiltration and immune-related pathways in PRAD cohorts (53, 54), but such associations do not prove that cuproptosis directly remodels immunity. More mechanistic support for copper-immunity crosstalk comes from studies showing that intratumoral copper can modulate PD-L1 expression and influence tumor immune evasion (55, 56), and from non-prostate models suggesting links among cuproptosis, WNT signaling, PD-L1 regulation, and CD8+ T-cell cytotoxicity (57, 58). These findings are biologically relevant because PCa often responds poorly to immune checkpoint inhibitors, but they should not be interpreted as evidence that copper ionophores or chelators will reliably sensitize unselected PCa patients to immunotherapy. Future PCa studies should directly measure DAMP release, dendritic-cell activation, T-cell priming, PD-L1 dynamics, myeloid-cell polarization, and antitumor efficacy after genetically or pharmacologically confirmed cuproptosis.

### 4.3 Crosstalk between cuproptosis and other cell death modalities

The multiple programmed cell death (PCD) pathways within cells are not isolated linear modules but form a complex, interconnected network, exhibiting extensive crosstalk. The relationship between cuproptosis and ferroptosis, which has received significant attention in recent years, is particularly noteworthy. Ferroptosis is an iron-dependent form of regulated cell death driven by lipid peroxidation, with the core biochemical event being the collapse of the cellular antioxidant system (particularly the glutathione peroxidase 4 (GPX4)/GSH system) and the catastrophic accumulation of lipid peroxides (59). Cuproptosis and ferroptosis intersect at multiple levels. First, both are closely related to mitochondrial function. Mitochondria are not only the core execution site of cuproptosis but also the primary location for reactive oxygen species (ROS) production and lipid peroxidation, making mitochondrial dysfunction a common feature of both death forms. Second, both are intricately regulated by GSH levels. GSH is not only a necessary substrate for GPX4 function but also directly chelates copper ions; thus, GSH depletion can simultaneously promote both ferroptosis and cuproptosis, making it a key node linking the two death forms. This intrinsic connection provides a solid theoretical foundation for designing combination therapeutic strategies capable of activating multiple cell death pathways concurrently. Recent studies indicate that some drugs or advanced nanomaterials can simultaneously target iron and copper metabolism, synergistically inducing ferroptosis and cuproptosis, thereby achieving an antitumor effect far exceeding that of single-pathway inducers. For instance, one study designed a nanozyme responsive to the TME that simultaneously releases iron and copper ions, triggering both death modalities within tumor cells to achieve highly efficient synergistic tumor killing (57). For tumors that have developed resistance to a single cell death mode (such as apoptosis or ferroptosis), combined induction of cuproptosis may become an effective “breaching” strategy. Given that ferroptosis has already demonstrated substantial potential in treating prostate cancer, particularly CRPC (5), in-depth exploration of the synergistic mechanisms and combined treatment regimens of cuproptosis and ferroptosis in prostate cancer is undoubtedly a highly promising research direction. Additionally, a potential link may exist between cuproptosis and apoptosis. Although the core mechanism of cuproptosis is defined as caspase-independent, under certain experimental conditions, high concentrations of copper ionophores inducing cuproptosis may also be accompanied by some apoptotic features, such as increased mitochondrial outer membrane permeability, cytochrome c release, and downstream caspase-3 activation (41). This may not be an inherent feature of cuproptosis but rather a consequence of severe copper-induced mitochondrial damage acting as a potent upstream stress signal that activates the classical mitochondrial apoptosis pathway in certain cellular contexts. This phenomenon suggests that under specific cellular backgrounds and stimulation intensities, cuproptosis may co-occur or interconvert with other death forms; the specific regulatory mechanisms and molecular switches warrant further in-depth investigation.

## 5 Key signaling pathways and epigenetic mechanisms regulating cuproptosis

The progression of prostate cancer is driven by a complex signaling network and a dynamically changing epigenetic landscape. Cuproptosis, as a downstream cell fate decision, has its triggering threshold and sensitivity profoundly influenced by these upstream core regulatory networks. This chapter will focus on two central signaling pathways in prostate cancer—androgen receptor (AR) signaling and PTEN deficiency/PI3K signaling—as well as how epigenetic modifications such as DNA methylation and non-coding RNAs finely regulate the occurrence of cuproptosis.

### 5.1 Interaction between androgen receptor signaling pathway and cuproptosis

The AR signaling pathway is the central axis driving the initiation, progression, and evolution of prostate cancer and remains active in many CRPC tumors (60, 61). A direct prostate cancer-specific link between AR biology and copper metabolism has been reported through AR-mediated regulation of SLC31A1/CTR1-dependent copper uptake (33). Whether AR directly controls the transcription of core cuproptosis effectors such as FDX1, DLAT, LIAS, or MTF1 in clinically relevant PCa states remains less clear. Functional evidence has recently strengthened this connection: in CRPC models, enzalutamide increased mitochondrial dependency and sensitized cells to copper ionophores such as elesclomol and disulfiram, with reported cuproptosis hallmarks including lipoylated protein aggregation and Fe-S cluster protein instability (41). This study provides an important preclinical rationale for combining AR pathway inhibition with copper ionophores, including in resistant models, but it should not be described as establishing clinical efficacy. The results were generated primarily in cell and xenograft systems, and the optimal patient subset, dosing schedule, copper exposure, safety profile, and companion biomarkers remain undefined. Accordingly, AR inhibition should be framed as a potential means of creating or amplifying mitochondrial/cuproptosis vulnerability, not as a direct or sufficient cuproptosis inducer. Future work should use ChIP-seq, chromatin accessibility profiling, CRISPR perturbation, and patient-derived CRPC organoids to define whether AR directly regulates the cuproptosis machinery and whether this regulation predicts response to copper ionophores.

### 5.2 PTEN deficiency and cuproptosis sensitivity

Phosphatase and tensin homolog (PTEN) is one of the most commonly inactivated tumor suppressor genes in prostate cancer, and its functional loss is a key driver of malignant progression, therapeutic resistance, and transformation towards CRPC (1). PTEN, a dual-specificity phosphatase, primarily functions as a lipid phosphatase, converting phosphatidylinositol (3–5)-trisphosphate (PIP3) to phosphatidylinositol (4, 5)-bisphosphate (PIP2), thereby potently antagonizing phosphatidylinositol 3-kinase (PI3K) activity and suppressing the downstream AKT/mechanistic target of rapamycin (mTOR) signaling pathway. The PI3K/AKT/mTOR pathway is a central hub regulating cell growth, proliferation, metabolism, and survival. The impact of PTEN deficiency on cuproptosis sensitivity is complex and multifaceted, potentially involving two opposing effects. On one hand, sustained activation of the AKT/mTOR pathway profoundly promotes glycolysis (the Warburg effect) and biosynthesis, which could reduce cellular dependency on mitochondrial OXPHOS, thus conferring a degree of resistance to cuproptosis. However, on the other hand, the AKT/mTOR pathway also profoundly influences mitochondrial function and biogenesis. For instance, mTOR activation can promote mitochondrial biogenesis and function to meet the immense energy demands of rapid cell proliferation. This enhancement of mitochondrial function and TCA cycle flux could, conversely, increase cellular sensitivity to cuproptosis, an attack directly targeting mitochondrial metabolism. Based on this, an intriguing yet unverified hypothesis is that in the CRPC stage, PTEN-deficient tumors might become more dependent on enhanced mitochondrial function for survival and proliferation, thereby becoming “paradoxically” sensitive to cuproptosis inducers. Furthermore, existing research has identified core metabolic regulatory pathways such as PI3K/mTOR and AMP-activated protein kinase (AMPK) as upstream regulators of cuproptosis, further suggesting that the status of the PTEN/PI3K/AKT axis likely serves as a critical checkpoint determining cuproptosis sensitivity. Future research needs to employ isogenic cell lines with PTEN knockout or knock-in, as well as patient-derived models with well-defined PTEN status, to precisely answer the key question: “Does PTEN deficiency promote or inhibit cuproptosis?” Elucidating the exact relationship between PTEN status and cuproptosis sensitivity will be of significant clinical guidance for achieving precision medicine in prostate cancer, particularly for selecting effective cuproptosis-targeting drugs for the large subgroup of patients harboring PTEN loss.

### 5.3 Epigenetic regulation of cuproptosis

Epigenetic modifications, including DNA methylation, histone modifications, and regulation by non-coding RNAs, heritably regulate gene expression without altering the DNA sequence and constitute a core mechanism in tumorigenesis and progression. The expression of cuproptosis-related genes is likewise tightly controlled by epigenetic regulation, which not only explains their expression heterogeneity in tumors but also provides a new avenue for tumor cells to evade cuproptosis and opens up novel therapeutic ideas for reversing resistance. In prostate cancer, hypermethylation of tumor suppressor gene promoters and hypomethylation of oncogenes are common epigenetic events (62). As the “master switch” of cuproptosis, FDX1 expression has been reported to be downregulated in various cancers (25), and recent studies indicate that this is largely due to DNA hypermethylation of its promoter region or the introduction of repressive histone modifications (e.g., H3K27me3). By epigenetically silencing FDX1, tumor cells can effectively turn off the “trigger” of cuproptosis, thereby acquiring resistance to copper ionophores. Conversely, using already approved epigenetic drugs such as DNA methyltransferase inhibitors (e.g., decitabine) or histone deacetylase inhibitors (HDACi) may reactivate the expression of key CRGs like FDX1, thereby “sensitizing” tumor cells and restoring their sensitivity to cuproptosis inducers. This strategy offers a novel, clinically feasible approach to overcome cuproptosis resistance. Long non-coding RNAs (lncRNAs) are a class of non-protein-coding RNAs over 200 nucleotides in length; they act as “fine-tuners” of gene expression and play crucial roles in virtually all pathological processes of prostate cancer (62). In recent years, an increasing number of studies have begun to focus on the role of lncRNAs in regulating programmed cell death, including cuproptosis (63). Bioinformatics analyses have preliminarily screened a series of cuproptosis-related lncRNAs and constructed prognostic models based on their expression profiles, demonstrating good predictive performance. These lncRNAs may regulate cuproptosis through various sophisticated mechanisms. For example, they can act as “molecular sponges” to competitively adsorb specific microRNAs (miRNAs), thereby relieving the miRNA-mediated translational inhibition of mRNAs of CRGs, including FDX1 and DLAT; or they can serve as “structural scaffolds” or “molecular guides,” recruiting chromatin-modifying complexes (such as Polycomb repressive complex 2 (PRC2) or lysine-specific demethylase 1 (LSD1)) to specific CRG loci to regulate their chromatin state and transcriptional activity. For instance, a hypothetical lncRNA-CuproReg might specifically recruit enhancer of zeste homolog 2 (EZH2), the catalytic subunit of PRC2, to the FDX1 promoter, leading to transcriptional silencing of FDX1 and resistance to cuproptosis (64). Identifying key lncRNAs that regulate cuproptosis in prostate cancer and elucidating their mechanisms of action will not only provide us with novel prognostic markers and potential therapeutic targets (e.g., using antisense oligonucleotide (ASO) technology to target these lncRNAs) but also greatly deepen our understanding of the complexity of the cuproptosis regulatory network.

## 6 Therapeutic strategies targeting cuproptosis in prostate cancer

**Figure 4:**
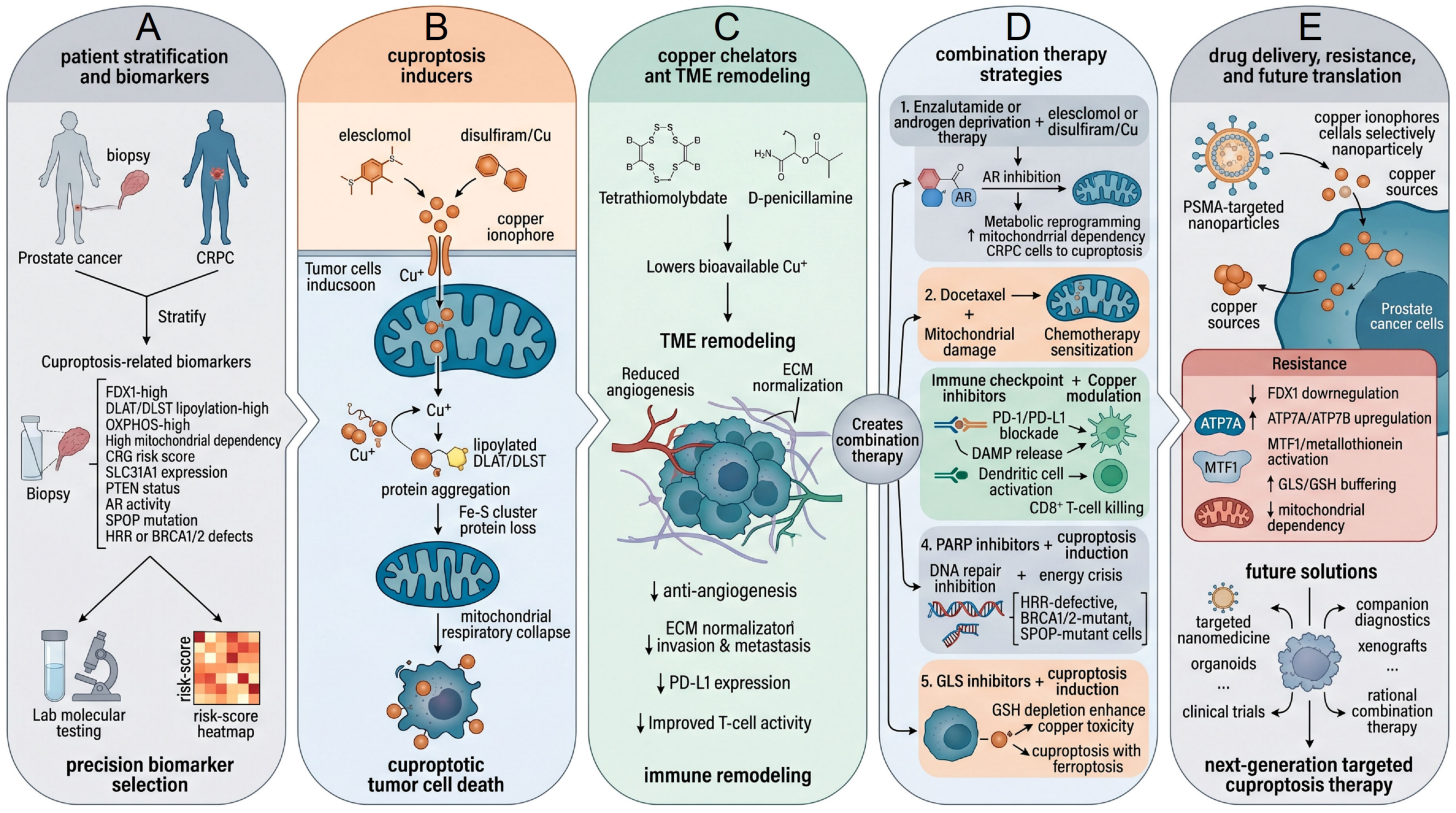
Therapeutic strategies targeting cuproptosis in prostate cancer. **(A)** Precision biomarker selection stratifies prostate cancer patients based on cuproptosis-related signatures (e.g., FDX1 status, DLAT/DLST lipoylation, and mitochondrial dependency) to identify optimal candidates for therapy. **(B)** Cuproptosis inducers, such as copper ionophores (elesclomol, disulfiram/Cu), shuttle copper into the mitochondria to trigger lipoylated protein aggregation, Fe-S cluster loss, and cuproptotic cell death. **(C)** Conversely, copper chelators induce systemic copper starvation, remodeling the tumor microenvironment through anti-angiogenesis, ECM normalization, and enhanced immune cell infiltration. **(D)** Rational combination strategies integrate cuproptosis modulation with standard-of-care therapies—including androgen receptor (AR) inhibition, chemotherapy, immune checkpoint inhibitors, PARP inhibitors, and GLS inhibitors—to induce synthetic lethality and overcome therapeutic resistance. **(E)** Future translational approaches highlight next-generation PSMA-targeted nanomedicine for specific drug delivery, alongside strategies to counteract acquired resistance mechanisms such as FDX1 downregulation or metallothionein activation.

The elucidation of cuproptosis has generated new therapeutic hypotheses for prostate cancer, particularly for subsets of CRPC with mitochondrial dependency. However, therapeutic targeting of cuproptosis in PCa remains investigational. Strategies can be broadly divided into copper ionophores or related approaches that increase bioavailable intracellular copper, and copper chelators that reduce systemic or tumor-associated copper. These approaches differ mechanistically and may produce antitumor effects through cuproptosis, oxidative stress, proteasome inhibition, cuproenzyme blockade, angiogenesis suppression, or immune modulation. Because copper is essential for normal physiology, both copper loading and copper depletion have narrow therapeutic windows; clinical development therefore requires careful dosing, monitoring, tumor-selective delivery, and validated biomarkers (**Figure 4**).

### 6.1 Antitumor potential of cuproptosis inducers

Cuproptosis inducers aim to increase bioavailable copper in tumor cells to levels that engage mitochondrial lipoylated proteins and trigger proteotoxic stress. Copper ionophores such as elesclomol and disulfiram/copper are useful experimental tools, but neither should be portrayed as ready for routine clinical use in PCa. Elesclomol can deliver copper to mitochondria and has shown potent activity in multiple preclinical models (65, 66), and prostate cancer cell-line data suggest nanomolar cytotoxicity in some contexts (67). However, cytotoxic potency *in vitro* does not guarantee a favorable therapeutic index in patients. The phase III SYMMETRY trial in advanced melanoma failed to improve progression-free survival in an unselected population and was stopped after an early overall-survival imbalance, although a subgroup with normal LDH appeared to benefit (68). This history underscores the need for metabolic and safety-based patient selection rather than broad clinical application. Disulfiram/copper also has prostate cancer-specific preclinical activity; in PC3 and DU145 cells, disulfiram/copper induced cytotoxicity and apoptosis-related effects. Nevertheless, disulfiram is rapidly metabolized, its active copper complex exposure is difficult to control, and its mechanisms include proteasome inhibition, ROS generation, and apoptosis in addition to possible cuproptosis. Novel formulations, nanoparticles, and PSMA-targeted delivery could theoretically widen the therapeutic window, but this remains preclinical. Before advancing cuproptosis inducers in PCa, studies should demonstrate canonical biomarkers of cuproptosis, define dose-limiting toxicities, and establish patient-selection criteria such as FDX1/lipoylation competence, SLC31A1 expression, copper-buffering capacity, and OXPHOS dependency.

### 6.2 Therapeutic applications of copper chelators

Copper chelators represent a mechanistically distinct strategy from cuproptosis induction. Rather than increasing mitochondrial copper to trigger cell death, chelators such as tetrathiomolybdate (TTM) and D-penicillamine lower bioavailable copper and may inhibit copper-dependent processes including angiogenesis, LOX-mediated extracellular matrix remodeling, and possibly PD-L1-associated immune evasion (69). Therefore, their antitumor effects should not be described as cuproptosis itself. In PCa, the evidence supporting chelators remains largely indirect or extrapolated, and the optimal patient population is unknown. Systemic copper depletion also carries risks, including hematologic, neurologic, hepatic, and nutritional toxicities, and may impair normal immune or mitochondrial function if excessive. Thus, chelation strategies require careful pharmacodynamic monitoring, such as serum copper/ceruloplasmin and potentially tumor copper imaging, and should be evaluated as microenvironment-modulating or anti-cuproenzyme approaches rather than as broadly cytotoxic therapies. Their combination with radiotherapy, chemotherapy, or immunotherapy remains rational but unproven in prostate cancer-specific clinical settings.

### 6.3 Combination therapy strategies: synergism and overcoming resistance

Combination therapy is the most plausible translational path for copper-targeting approaches in PCa, but most proposed combinations remain hypotheses. For AR-directed therapy, preclinical evidence indicates that enzalutamide can increase mitochondrial dependency and sensitize CRPC cells to copper ionophores such as elesclomol and disulfiram, with *in vitro* and xenograft activity (41). This finding is important, yet it should be presented as preclinical rationale rather than as a validated treatment for enzalutamide-resistant patients. With chemotherapy, copper ionophores might enhance mitochondrial damage or stress responses, but prostate cancer-specific synergy with docetaxel has not been rigorously demonstrated across resistant models or in patients. With immunotherapy, copper modulation may affect PD-L1 and immune-cell function, and cuproptosis might release immunostimulatory signals; however, there is no clinical evidence that copper ionophores or chelators overcome the generally low response rate of unselected PCa to immune checkpoint inhibitors. Similarly, combining cuproptosis inducers with PARP inhibitors in HRR-deficient or SPOP-mutant disease is a biologically plausible synthetic-lethal concept, but direct evidence is currently absent. Future combination studies should incorporate mechanistic pharmacodynamic endpoints, such as DLAT/DLST aggregation, Fe-S cluster protein loss, mitochondrial respiration changes, copper accumulation, and immune remodeling, rather than relying only on tumor-growth inhibition. Clinical translation should proceed only with clear dose-escalation plans, toxicity stopping rules, and companion biomarker strategies.

### 6.4 Clinical research status and challenges

Clinical translation of cuproptosis-targeting drugs in prostate cancer remains at an early and uncertain stage. Most clinical experience with copper ionophores or copper-modulating drugs comes from non-prostate tumors, and prostate cancer-specific trials are limited. The clearest prostate cancer clinical caution comes from the phase Ib study of disulfiram plus copper in mCRPC: the study did not establish meaningful clinical benefit, reported toxicity, and highlighted pharmacokinetic limitations of oral disulfiram/copper exposure (42). Likewise, the failure of elesclomol plus paclitaxel in an unselected phase III melanoma population demonstrates that copper-ionophore activity can be strongly context-dependent and clinically limited by patient selection and safety (68). These data argue against implying near-term clinical applicability in PCa without rigorous biomarker-driven trials. Several barriers must be addressed. First, no validated companion diagnostic currently identifies PCa patients likely to respond to cuproptosis induction; candidate markers such as FDX1, LIAS/LIPT1, DLAT/DLST lipoylation, SLC31A1, ATP7A/B, metallothioneins, GSH-buffering capacity, and OXPHOS dependency require analytical and clinical validation. Second, copper has a narrow therapeutic window, and both copper loading and copper depletion can injure normal tissues; dose, schedule, copper supplementation, chelation rescue, and organ-specific monitoring must be optimized. Third, negative and inconclusive clinical data should be incorporated into trial design rather than treated as historical artifacts. Fourth, resistance mechanisms may include FDX1 downregulation, impaired protein lipoylation, increased copper efflux, metallothionein/MTF1 activation, enhanced glutathione buffering, or a shift toward glycolysis. Accordingly, future PCa trials should be small, biomarker-enriched, pharmacodynamically intensive, and designed to confirm that canonical cuproptosis occurs in tumor tissue before efficacy claims are made.

## 7 Conclusions and perspectives

Since its formal definition in 2022, cuproptosis has become an active area of cancer biology, but its role in prostate cancer remains incompletely defined. This review highlights a biologically coherent connection among copper homeostasis, mitochondrial metabolism, AR signaling, the immune microenvironment, and therapeutic resistance. At the same time, it emphasizes that much of the current PCa evidence is indirect, retrospective, bioinformatic, preclinical, or extrapolated from other tumor types. Therefore, cuproptosis should be regarded as a promising research framework and potential therapeutic vulnerability, not yet as a clinically validated target for prostate cancer.

Taken together, research on cuproptosis in prostate cancer has produced several important but preliminary advances. Bioinformatic studies suggest that CRG expression patterns may correlate with prognosis and immune infiltration, while preclinical work indicates that AR pathway inhibition can increase mitochondrial dependency and sensitize CRPC models to copper ionophores. However, the number of direct prostate cancer functional studies remains small, and clinical evidence is limited and partly negative or inconclusive. Thus, the field now needs rigorous prostate cancer-specific validation rather than broader claims about therapeutic promise.

However, looking ahead, the field still faces numerous key scientific questions to be answered and technical hurdles to be overcome, and these challenges precisely constitute the opportunities and directions for future research.

First, deepen the precise analysis of the regulatory mechanisms of cuproptosis in prostate cancer. The current core challenge lies in translating findings from pan-cancer studies into prostate cancer-specific knowledge. For example, how exactly do the AR signaling pathway and PTEN deficiency status precisely regulate the transcription and translation of core genes like FDX1 and DLAT? What is the spectrum of DAMPs released upon cuproptosis induction in prostate cancer cells, and what is its real efficiency in activating antitumor immunity *in vivo*? We need to move from bioinformatics predictions to rigorous functional validation based on prostate cancer cell lines, organoids, and genetically engineered mouse models.

Second, focus on prostate cancer heterogeneity and explore subtype-specific cuproptosis sensitivity. Prostate cancer is a highly heterogeneous tumor, and different molecular subtypes (e.g., ERG fusion, SPOP mutation, PTEN deficiency) may exhibit vastly different sensitivities to cuproptosis. Future research should integrate single-cell multi-omics techniques to systematically map the “cuproptosis sensitivity landscape” of different prostate cancer subpopulations and identify the key molecular drivers determining these differences. For instance, does SPOP mutation indirectly regulate the cuproptosis pathway by affecting protein stability? This is crucial for achieving truly personalized cuproptosis therapy.

Third, develop and validate precise biomarkers and highly efficient next-generation targeted drugs. The combined therapy study with enzalutamide suggests that the ideal biomarker may not only be the expression level of FDX1 but should also include functional indicators assessing the mitochondrial dependency of tumor cells. This points the way for the development of companion diagnostics. Concurrently, drug development should go beyond existing copper ionophores and leverage artificial intelligence-assisted drug design, proteolysis-targeting chimera (PROTAC) technology, or targeted nano-delivery systems to develop a new generation of copper metabolism modulators with higher tumor targeting, controlled copper release kinetics, and lower off-target toxicity, aiming to widen the therapeutic window and maximize efficacy.

Fourth, promote biomarker-driven clinical trials grounded in solid biological evidence. Future clinical trial designs should avoid broad, unselected enrollment. Based on the 2024 preclinical data, combinations such as enzalutamide with elesclomol or disulfiram may be worth testing only after adequate dose-finding, pharmacokinetic optimization, toxicity monitoring, and biomarker enrichment. Candidate biomarkers should include FDX1 and protein lipoylation status, copper transporter expression, mitochondrial dependency, and pharmacodynamic evidence of DLAT/DLST aggregation or Fe-S cluster protein loss. Window-of-opportunity studies may help determine whether cuproptosis can actually be induced in human PCa tissue and whether it measurably remodels the immune microenvironment.

In summary, cuproptosis offers a new mechanistic lens through which to study prostate cancer metabolism and copper vulnerability. Its translational value will depend on whether future studies can demonstrate canonical cuproptosis in PCa tissues, identify the right molecularly selected patients, and deliver copper-modulating agents safely within a narrow therapeutic window. Rather than a ready-made therapeutic weapon, cuproptosis should be viewed as a carefully testable opportunity for precision oncology in advanced PCa and CRPC. Progress in this area will require prostate cancer-specific models, prospective biomarker validation, transparent reporting of negative data, and rational early-phase trials with strong pharmacodynamic endpoints.
